# Developmental Changes in Memory-Related Linguistic Skills and Their Relationship to Episodic Recall in Children

**DOI:** 10.1371/journal.pone.0137220

**Published:** 2015-09-02

**Authors:** Izumi Uehara

**Affiliations:** Department of Psychology, Ochanomizu University, Bunkyo-ku, Tokyo, Japan; Birkbeck College, UNITED KINGDOM

## Abstract

This longitudinal study of nine children examined two issues concerning infantile amnesia: the time at which memories for events experienced before the age of 3–4 years disappear from consciousness and whether this timing of memory loss is related to the development of specific aspects of episodic and autobiographical memory. This study followed children from infancy to early childhood and examined the central role of three verbal–cognitive milestones related to autobiographical memory: the age at which children begin to report autobiographical memories using the past tense (Milestone 1); the age at which they begin to verbally acknowledge past events (Milestone 2); and the age at which they begin to spontaneously use memory-related verbs (Milestone 3). As expected, memories of events that occurred before 3–4 years of age were affected by infantile amnesia. Achievement of these milestones followed almost the same developmental progression: Milestone 1 (1 year; 10 months (1;10) to 3 years; 4 months (3;4)) was followed by Milestones 2 (3;1 to 4;0) and 3 (3;5 to 4;4). Milestone 2 was typically related to the onset of infantile amnesia, whereas Milestone 1 occurred during the period for which the children became amnesic as they aged. These data suggest that linguistic meta-cognitive awareness of personal memory is the key feature in infantile amnesia.

## Introduction

The scarcity of autobiographical memories in humans from the period prior to the age of 3 to 4 years is called “infantile amnesia” [1]. This phenomenon has been extensively documented in both adults [2–4] and elementary school children [5–7]. It is clear that this type of amnesia cannot be attributed to encoding deficits given that healthy infants and 2–3-year-old children possess relatively good memories [8–10]. An accelerated forgetting curve during childhood has been proposed as one reason for poor childhood memory [5, 11–13], but the adult inaccessibility of memories before 3–4 years of age is not well explained by this forgetting curve. Mechanisms underlying infantile amnesia and related important issues are yet to be clarified; for example, why are the earliest experiences that both adults and children recall the ones that occurred around 3–4 years rather than at 0 or 1 year of age, and what cognitive abilities are related to this amnesia?

Two significant questions concerning infantile amnesia can be posed here. The first concerns the timing of the inability to recall events experienced before 3–4 years of age in the context of individual development: When do originally retained memories for events experienced before 3–4 years of age begin to disappear from consciousness? The second question concerns the relationship with other abilities: Is this timing of memory loss related to the development of specific aspects of episodic and autobiographical memory? The present study focuses on the development of memory-related linguistic abilities as specific aspects of autobiographical memory. Past literature has discussed the relationships among the development of autobiographical memory, infantile amnesia, and the emergence of cognitive abilities related to narrative form, self-concept, theory of mind, and language skills; such relationships have been inferred based on relationships between young children’s autobiographical recall and the development of these abilities [14–18]. However, data pertaining to the relationship between the onset of infantile amnesia and the development of such cognitive abilities are lacking. Thus, this study aimed to address this existing gap using a longitudinal method aligned with the general approach described above. Before explaining in detail the purpose of this study, I will briefly review longitudinal studies on infantile amnesia and findings on the development of linguistic aspects of autobiographical memory (i.e., narrative).

Much of the longitudinal research on the onset of infantile amnesia in young children has examined children’s memory for single- or multiple-instance events selected by researchers or mothers by asking the children about them repeatedly. For example, Peterson and colleagues [19–21] examined children’s recall for single-instance traumatic injuries experienced between the ages of 13 and 34 months by asking each child to verbally recall the event on multiple occasions up to the age of 60 months. Solter [22] examined one child’s recall of a surgery experienced at the age of 5 months by asking the child about it at 29 months and at 40 months. Cleveland and Reese [23] examined the recall of 65-month-old children for non-traumatic early life events. Prior to testing, mothers had been instructed to select specific events that had occurred just a few months prior to each session and discuss them with their children during four separate sessions when the children were 19–40 months of age. At 65 months (testing), the children were asked about these events. These studies are informative, but repeated previous discussion about the target event may have enabled children and/or mothers to predict that the child would be tested on his or her memory for the event, which may have influenced the results. Moreover, the past literature has overlooked aspects of infantile amnesia involving loss of memory for many ordinary experiences.

A number of studies have examined the development of autobiographical memory in relation to its linguistic aspects, especially narratives. The first detailed analysis of the structures and content of a child’s utterances in relation to narrative development dates back to Nelson’s report on “Emily” [24]. Based on the utterance data obtained during pre-bed discussions with her parents when she was 21 to 36 months of age, this report first suggested the role of narratives in the development of autobiographical memory. Successive studies have suggested that the development of the ability to form narratives is an important component of a child’s ability to recall past events later in life [15, 25], often by demonstrating that the children of parents who speak in greater detail about past events are better able to provide coherent narratives about their own past [26–28]. However, very few studies have examined children’s own memory-related language use and its relationship to autobiographical reports [29] despite the suggested relationship between parental talk about memories in the presence of children and children’s own memories about early life [27, 28, 30]. The development of children’s memory-related linguistic abilities and its relationship to the development of autobiographical memory has never been explored.

It can be assumed that some meta-cognitive abilities have to be in place before a child is able to remember and report an autobiographical memory. The present study treated memory-related linguistic abilities as measurable and informative indicators of meta-cognitive development. Using longitudinal methods, the present study aimed to investigate whether there is a relationship between infantile amnesia and certain memory-related linguistic milestones in autobiographical memory. Specifically, this study examined the question of when originally retained memories for events experienced before 3–4 years of age disappear from consciousness (i.e., the onset of infantile amnesia) and whether the onset of infantile amnesia is related to children’s ability to verbally acknowledge past events (assumed to occur typically between the ages of 3 and 4 years [31]). This ability does not simply reflect language proficiency. Rather, it is an indication of a specific linguistic skill related to the development of autobiographical memory. In addition to exploring the mechanisms underlying infantile amnesia, the present study also aimed to examine whether there is a specific developmental progression of memory-related language skills among children.

To determine the critical boundary before which previous personal memories cannot be recalled (i.e., the boundary of infantile amnesia), children were tested at regular intervals about their memory for events that occurred in their distant and immediate past. Moreover, I identified three developmental milestones that are associated with memory-related language use and that are hypothesized to be intimately related to the onset of infantile amnesia.

*Milestone 1*: *first episodic report*. This refers to the age at which a child first provides a report of an event in his or her past using the past tense [29] without merely repeating the words used by the researcher or mother.

*Milestone 2*: *first verbal recognition*. This refers to the age at which a child is able to verbally distinguish between items that he or she has seen before and items that he or she has not seen before.

*Milestone 3*: *memory-verb acquisition*. This refers to the age at which a child begins to spontaneously use the verbs “remember” and/or “forget” appropriately.

Through cognitive testing of a small subset of the children [32], I previously noticed a possibly fixed temporal relationship between Milestones 1 and 2, though it was statistically unsupported. Also, in a preliminary report, I established the current method for identifying Milestone 3 in children, hoping to discern its temporal relationship with other developmental milestones in future studies [33]. Although the data at these research stages were suggestive of a typical developmental order such that Milestone 1 would typically appear earlier, more intricate aspects of the true developmental order, especially the order of Milestones 2 and 3, remained ambiguous. The present study aims to provide valid statistical evidence identifying the temporal relationships, if any, among these cognitive skills in children. Adopting a longitudinal approach, a method that has not been used in this particular domain thus far, it will compare the age at onset of each milestone with the onset of infantile amnesia. To reliably identify the onset of infantile amnesia, the present study focused on memory about past episodic experiences corresponding to a single personal event. The choice of this particular type of memory was based on my preliminary examination of a few of the current participants, who had participated in analyses of various memory types, including memory for repeated experiences, nonverbal procedural memory about physical experiences, and false memory [34]. This preliminary examination confirmed that these latter types of memory were much more vulnerable than memories for single events to various undesirable effects.

## Methods

### Participants

Nine middle-class Japanese-speaking children and their mothers participated in this study. All were residents of cities neighboring Tokyo and were from two-parent families. The sex, age at participation, and number of sessions in which each child participated are presented (Table 1). Participant YA’s participation was shorter than that of the others because he was no longer available after his family moved when he was 5 years and 0 months of age (5;0). Written consent was obtained from the mothers of all children, and all procedures in this study were approved by the Humanities and Social Sciences Research Ethics Committee, Ochanomizu University (Ethics approval number: 2013–25).

**Table 1:** Sex, age at participation, and the number of sessions for each child who participated.

Child	Sex	Ages	Number of sessions
HM	Boy	From 1 y, 5 m to 6 y, 4.5 m	22
TS	Boy	From 0 y, 10.5 m to 6 y, 6.5 m	24
KO	Boy	From 1 y, 10 m to 6 y, 5 m	22
YA	Boy	From 2 y, 5 m to 4 y, 11 m	13
KS	Boy	From 3 y, 0 m to 6 y, 10 m	20
KN	Girl	From 1 y, 5 m to 6 y, 8.5 m	20
SA	Girl	From 2 y, 1 m to 8 y, 1 m	21
AH	Girl	From 1 y, 6 m to 6 y, 7.5 m	20
MH	Girl	From 1 y, 7 m to 6 y, 6.5 m	25

### Materials

Assessments of developmental changes were based on information obtained via a questionnaire [32] focused on the children’s memory and language use. This questionnaire had two sections; the first section included three yes–no questions:

1. Have you ever heard your child say the word "remember”?
2. Have you ever heard your child say the word "forget”?
3. Have you ever heard your child talk about his/her own experiences using the past tense?

The mother was asked to answer “Yes” or “No” to each question and then to record concrete examples in support of affirmative answers. In the second section of the questionnaire, the mother was asked to record and date events occurring during the months-long period between interview sessions that deviated from the child’s daily routine (see S1 Appendix). Some of these events were used in probes of the child’s autobiographical memory in the longitudinal recall tasks in later interviews.

The present study used a total of 240 crayon drawings depicting simple colored figures that are easily identified by children as memory items. Each interview used 20 individual drawings and no drawing was used more than once.

A variety of toys was always available during each interview to enrich the environment, and each child was encouraged to play with these toys. In addition to the mother’s event log, a portion of the time each child spent playing with specific toys was later used as a target event in autobiographical memory recall tasks.

### Procedure

Interviews were conducted with each child–mother pair approximately every 2–3 months, with a range of 1.0–4.5 months (except that KN’s interview was once delayed by 6.5 months). After the age of 5;5, when HM, TS, SA, AH, and MH became busy with other activities (piano, swimming lessons, and so on), several interviews with these children were conducted, approximately every 6 months. Before each interview, the mother completed the questionnaire and maintained a log of notable events.

Each meeting with the child and the mother typically lasted 1–3 hours (including short breaks for bathroom time and beverages). An interview of this length was necessary primarily because the children wanted to prolong the play-oriented portion of the interaction. Children very rarely became fretful or fussy by the end of the session, and no child was reluctant to interact with the experimenter in any interview.

The location of interviews was based on the convenience of the participants; interviews with HM, KO, SA, and AH were conducted at a university laboratory; interviews with YA (the first two), KN, and MH were conducted at their homes; and interviews with TS, YA (the remaining interviews), and KS were conducted at the experimenter’s personal office. Every meeting with each child–mother pair had the same structure. The mother gave the questionnaire and log to the experimenter who quickly reviewed the log and conducted a play-oriented interview with the child, which was followed by an interview with the mother. The mother remained in the room during the entire session, but she was instructed not to answer questions posed by the experimenter or the child unless she was asked to help.

During the play-oriented interview with the child, the experimenter used exciting games and attractive and unusual toys to engage the child. To facilitate talk about past events, the experimenter asked the child about previous personal events, including events and activities that occurred during previous meetings with the experimenter and the events included on the log provided by the mother. After the child’s interview, the mother was interviewed to confirm the accuracy of information provided by the child as well as to clarify answers that the mother provided on the questionnaire. Interviews were recorded on videotape and audiotape for off-line transcription and analysis with the permission of the mother.

### Assessment of the three milestones

The age of each child at each of the three memory-related language milestones was identified based on data from the interviews and questionnaires.

#### Determination of Milestone 1: age at first episodic report

The age at first episodic report (Milestone 1) was assessed with a combination of interview and questionnaire data. Reports were counted as valid only if children described distinct actual episodes using the past tense [29]. For instance, “I went to the zoo,” “I broke a water balloon,” and “I took a super-express train (with) Mom, Brother” were regarded as episodic reports whereas “Brother went to school” and “I saw Teacher” were not. In addition to the dialogue reported on the questionnaire, the child’s video-recorded dialogue from each interview was also examined to identify episodic reports. When the mother indicated in the questionnaire that the child had produced an episodic report, the experimenter asked the mother to elaborate on the event to ensure that it was a genuine case in which the child reported an episode rather than a case in which the child simply repeated the words used by others.

In all cases, the mothers reported through questionnaires that their children had passed this milestone before the behavior was observed during interviews. Due to this inevitable time lag between the mother’s testimony and the interview marking the attainment of a milestone, Milestone 1 was identified as the period between the first episodic report noted by the mother and the first episodic report observed during an interview.

#### Determination of Milestone 2: age at first verbal recognition

The age at first verbal recognition (Milestone 2) was assessed during the interview with each child via a memory task consisting of three phases: a learning phase immediately followed by two test phases. Because the purpose was to examine whether the child could understand and properly respond to recognition questions, the interval between the learning and test phases was purposefully kept short so that the task could measure the child’s ability to make a verbal judgment about recognition without possible confounds of decay and forgetting attributable to memory storage degradation. The start age of this task for each child is provided (see Results).

In the learning phase, the child was twice shown a series of 10 drawings and was then asked to name the object in the drawing. If the child failed to complete this task, the experimenter said the name and asked the child to repeat it. In the first memory-testing phase, the child was randomly presented with five pairs of drawings with each consisting of an old item and a new item. The child was asked to choose the old item according to a forced-choice paradigm. The second memory-testing phase used the five remaining old items as well as another five new items. These 10 drawings were randomly shown to the child one at a time, and the child was asked to say “Yes, I saw it” or “No, I did not see it” in response to each. It took approximately 11 minutes (3 minutes for the learning phase, 5 minutes for the retention interval, and 3 minutes for the testing phase) to complete each session. For six young children within certain age ranges (until 2;2 for AH, 2;5 for KN, and 2;9–11 for SA, MH, KO, and YA), the number of items to be remembered was decreased to six in the learning phase, and three of these were then mixed with three new items in each test phase because it was difficult for these children to pay attention to 10 items at these ages.

To be considered as having reached Milestone 2, a child had to choose the target drawing in all five drawing pairs in the first memory test and correctly answer “Yes” or “No” to nine of the 10 single drawings in the second memory test during the same interview session. The use of the combination of tests to determine this milestone relates to the concern that a single measure may be insufficient as a reliable index of passing this milestone because young children can show a novelty bias in a forced-choice paradigm and a bias toward answering “Yes” in a “Yes–No” paradigm [35, 36].

#### Determination of Milestone 3: age at memory-verb acquisition

The age at memory-verb acquisition (Milestone 3) was also assessed based on interview and questionnaire data. As was the case for Milestone 1, only utterances that were not simply the child repeating statements from the mother or the experimenter were counted. An independent coder listened to audio clips and judged whether a memory verb (“remember” or “forget”) was indeed used and whether it was used correctly; no disagreements between the experimenter and the coder occurred. Unlike Milestone 1, Milestone 3 focused on when a child first used memory verbs in interactions with the mother and the interviewer. (The one exception to this rule was YA, who did not use any memory-related verbs until the last interview but reportedly used such verbs with the mother more than a year earlier; in this case, the mother’s report was taken as empirical evidence.)

### Assessment of longitudinal recall

During each interview, the experimenter asked the child about experiences during previous interviews as well as about those described by his/her mother on the questionnaire. As in the assessment of the three milestones, the verbal component was crucial; that is, the child had to be able to talk about memories of events. Two types of events were probed: experimenter-initiated events and events noted by the mother.

Experimenter-initiated events occurred during the child’s interview. To create experiences that would be later used as the foci of probes, the experimenter and child played new and unique games during every interview. For example, in one session, the child and experimenter played a game in which the child was asked to identify an object in an opaque box by putting his or her hand through a decorated opening of the box to touch the object. Each game was usually played once so that a child’s memory of a game could refer only to that one particular experience. Data from games played more than once and from games that the mothers claimed the children played before or after an interview session were excluded from the analysis.

Other types of events used in this assessment were non-routine occurrences noted by the mother in her event log. Examples of such events included a head injury sustained at the nursery school followed by a hospital visit, playing with a snake when a petting zoo visited the nursery school, building a snowman with family members, and boarding a boat for the first time. To ensure that each experience corresponded with a single event, events that occurred multiple times were excluded from the analysis. Events that were videotaped and watched by the children after they actually experienced them were also excluded.

During every interview session, the experimenter asked children whether they remembered randomly selected events included in the current and previous logs. If they did, they were asked to provide details about the event. As expected, children were less likely to acknowledge having experienced an event or to provide related details successfully without a helpful prompt [16, 37]. Thus, following the method used in previous studies [19, 22, 23], children were gradually supplied with several cues when they did not answer the question. Although the majority of cues about past events were purely verbal, the experimenter occasionally used objects such as toys and tools as cues. A past event was judged to have been remembered when the child was able to add at least one new (and accurate) detail about the event without including any erroneous details. The events referenced were selected randomly and, thus, several events were tested more than once during the participation period. For example, a specific event experienced by a child at 2;5 was asked about at 2;8 and 3;3. Due to the interview schedule, most events were inquired about following an interval of 1 or more months, and these events were analyzed. The start age of this assessment and the number of events asked about in each session for each child are provided (Tables 2 and 3). Children were asked about approximately 2–5 events until 3 years of age, 6–7 events at 3–4 years of age, and 7–9 events after 4 years of age.

**Table 2:** Number of past events addressed during each session for four children.

HM						TS					
	Number of events asked						Number of events asked				
	The date of events						The date of events				
Age	–M1	M1–M2	M2–M3	M3–	Total	Age	–M1	M1–M2	M2–M3	M3–	Total
2 y, 0 m	2				2	2 y, 0 m	3				3
2 y, 2 m	2				2	2 y, 2 m	3				3
2 y, 4 m	2				2	2 y, 4 m	3				3
2 y, 6 m	4				4	2 y, 6.5 m	5				5
2 y, 8 m	1	4			5	2 y, 8.5 m	6				6
2 y, 11 m	1	4			5	2 y, 10.5 m	3	3			6
3 y, 2 m	2	4			6	3 y, 1.5 m	2	4			6
3 y, 5 m	4		3		7	3 y, 4.5 m	3	3			6
3 y, 7 m	4		4		8	3 y, 7.5 m	2	4			6
3 y, 9 m	2		5		7	3 y, 10.5 m	2	5			7
3 y, 11 m	2		4	1	7	4 y, 2 m	5		2		7
4 y, 1.5 m	3		2	2	7	4 y, 4 m	3		6		9
4 y, 4 m	3		1	3	7	4 y, 7.5 m	4		2	1	7
4 y, 7 m	3		3	2	8	4 y, 11 m	3		4	2	9
4 y, 10 m	1		3	3	7	5 y, 2.5 m	3		0	4	7
5 y, 1 m	2		1	4	7	5 y, 7 m	3		3	3	9
5 y, 5 m	4		2	2	8	6 y, 0 m	2		5	2	9
6 y, 0 m	1		0	8	9	6 y, 6.5 m	3		1	4	8
6 y, 4.5 m	1		2	4	7						
KO				YA							
	Number of events asked				Number of events asked						
	The date of events				The date of events						
Age	–M1	M2/M3–	Total	Age	–M1	M1–3 y, 6.5m		3 y, 6.5m–M2		M2–	Total
2 y, 6 m	3		3	2 y, 5 m	3						3
2 y, 9 m	1		1	2 y, 7 m	3						3
3 y, 0 m	2		2	2 y, 9 m	3						3
3 y, 2 m	3		3	2 y, 11.5 m	6						6
3 y, 5 m	4		4	3 y, 4 m	6						6
3 y, 7 m	4		4	3 y, 6.5 m	3	3					6
3 y, 10 m	5		5	3 y, 9.5 m	2	1		3			6
4 y, 0 m	6		6	3 y, 11.5 m	2	3		2			7
4 y, 2.5 m	5	1	6	4 y, 2.5 m	5			2			7
4 y, 5 m	3	5	8	4 y, 4 m	4			5			9
4 y, 7 m	3	4	7	4 y, 6 m	3			5			8
4 y, 10 m	2	5	7	4 y, 8 m	5			4			9
5 y, 1 m	3	5	8	4 y, 11 m	4			2			6
5 y, 5 m	2	7	9								
5 y, 9 m	1	7	8								
6 y, 1 m	2	7	9								
6 y, 5 m	1	7	8								

*Notes*.–M1: Number of addressed events that the child had experienced before the age at Milestone 1 (the interview at which the first episodic report was observed). M1–M2: Number of addressed events that the child had experienced on or after Milestone 1 but before Milestone 2. M2–M3: Number of addressed events that the child had experienced on or after Milestone 2 but before Milestone 3. M3–: Number of addressed events that the child had experienced on or after Milestone 3. M2/M3: Number of addressed events that the child had experienced on or after Milestones 2 and 3. (The ages at Milestones 2 and 3 were the same.) M1–3y, 6.5m: Number of addressed events that the child had experienced on or after Milestone 1 but before 3y, 6.5m. 3y, 6.5m–M2: Number of addressed events that the child had experienced on and after 3y, 6.5m but before Milestone 2. M2–: Number of addressed events that the child had experienced on or after Milestone 2. Total: Total number of past events addressed during the session.

**Table 3:** Number of past events addressed during each session for five children.

KS					KN						SA				
	Number of events asked					Number of events asked						Number of events asked			
	The date of events					The date of events						The date of events			
Age	–M1	M1–M2	M2–	Total	Age	–M1	M1–M2	M2–M3	M3–	Total	Age	–M2	M2–M3	M3–	Total
3 y, 0 m	5			5	1 y, 10 m	2				2	2 y, 1 m	2			2
3 y, 2 m	5			5	2 y, 0.5 m	2				2	2 y, 3.5 m	1			1
3 y, 4 m	4	2		6	2 y, 3 m	1	1			2	2 y, 7 m	2			2
3 y, 7 m	1	5		6	2 y, 5 m	0	4			4	2 y, 9 m	2			2
3 y, 9 m	2	4		6	2 y, 7.5 m	1	3			4	2 y, 10.5 m	2			2
3 y, 11 m	5		1	6	2 y, 10.5 m	1	5			6	2 y, 11.5 m	3			3
4 y, 1 m	5		2	7	3 y, 1 m	0	5			5	3 y, 2 m	6			6
4 y, 3 m	4		4	8	3 y, 3 m	6		0		6	3 y, 4 m	5	1		6
4 y, 5 m	3		4	7	3 y, 5.5 m	4		2		6	3 y, 6.5 m	4	2		6
4 y, 7 m	5		4	9	3 y, 10 m	3		0	6	9	3 y, 8.5 m	4	2		6
4 y, 10 m	2		7	9	4 y, 4.5 m	3		2	4	9	3 y, 11 m	3	3		6
5 y, 0 m	2		7	9	4 y, 8.5 m	3		1	5	9	4 y, 1.5 m	6	1		7
5 y, 2 m	1		8	9	5 y, 0 m	3		1	4	8	4 y, 5.5 m	6	1	0	7
5 y, 4 m	2		6	8	5 y, 4 m	2		2	5	9	4 y, 8.5 m	2	6	1	9
5 y, 7 m	2		5	7	5 y, 9 m	1		1	6	8	5 y, 1 m	2	4	3	9
5 y, 10 m	3		6	9	6 y, 3.5 m	1		0	8	9	5 y, 6 m	4	1	2	7
6 y, 1 m	1		6	7	6 y, 8.5 m	1		1	9	11	6 y, 0 m	3	2	2	7
6 y, 4 m	1		8	9							6 y, 6.5 m	2	0	7	9
6 y, 7 m	0		8	8							7 y, 0 m	4	0	5	9
6 y, 10 m	3		6	9							7 y, 4 m	3	1	4	8
											8 y, 1 m	3	0	5	8
AH					MH										
	Number of events asked					Number of events asked									
	The date of events					The date of events									
Age	–M1	M1–M2	M2–	Total	Age	–M1	M1–M2	M2–M3	M3–	Total					
2 y, 0 m	2			2	2 y, 0.5 m	2				2					
2 y, 2 m	2			2	2 y, 2.5 m	2				2					
2 y, 5 m	3			3	2 y, 4.5 m	3				3					
2 y, 7 m	4			4	2 y, 6 m	2				2					
2 y, 11 m	5			5	2 y, 8 m	2				2					
3 y, 2 m	4	2		6	2 y, 10.5 m	4				4					
3 y, 6 m	1	5		6	3 y, 1 m	5				5					
3 y, 8.5 m	3	3		6	3 y, 3 m	2	3			5					
3 y, 11 m	4		2	6	3 y, 5 m	1	5			6					
4 y, 1.5 m	4		3	7	3 y, 7 m	3	3			6					
4 y, 5.5 m	4		4	8	3 y, 9 m	4		2		6					
4 y, 8.5 m	3		6	9	3 y, 11 m	2		5		7					
4 y, 11.5 m	4		5	9	4 y, 1 m	3		2	2	7					
5 y, 2 m	1		8	9	4 y, 4 m	3		4	1	8					
5 y, 4 m	3		5	8	4 y, 6.5 m	2		4	1	7					
5 y, 9 m	4		4	8	4 y, 10 m	2		1	6	9					
6 y, 1 m	1		8	9	5 y, 2 m	2		1	6	9					
6 y, 7.5 m	0		7	7	5 y, 5 m	1		1	6	8					
					5 y, 9 m	1		0	7	8					
					6 y, 2.5 m	3		0	6	9					
					6 y, 6.5 m	1		0	7	8					

*Notes*. The abbreviations are the same as those used in Table 2. A new abbreviation,–M2, indicates the number of events addressed that the child had experienced before the age at Milestone 2.

## Results

### Milestone 1: age at first episodic report

**Fig 1:**
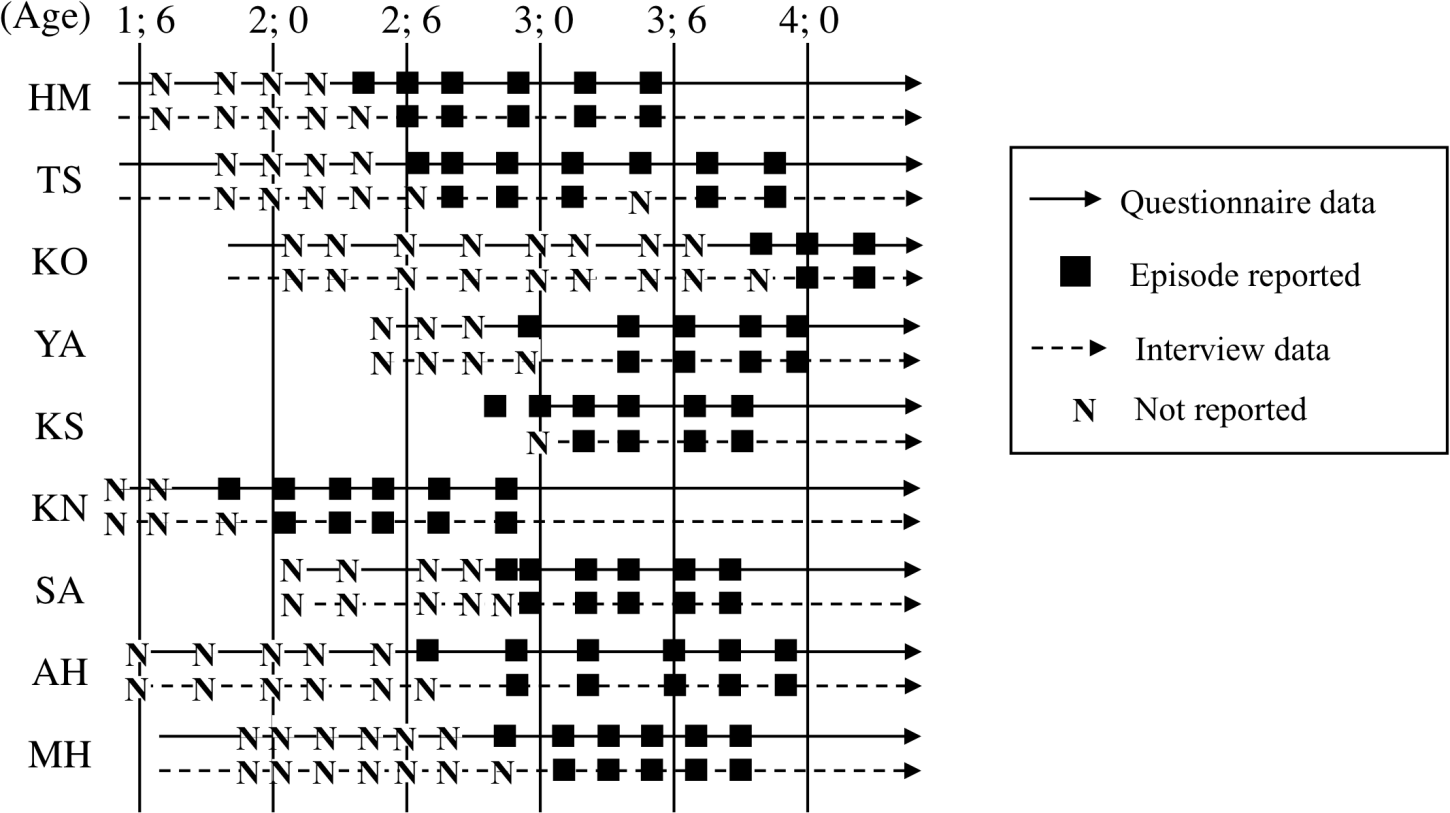
Episodic reports using the past tense in the questionnaire and interview data. “■” indicates that the child reported an actual episode and “N” indicates that no episodic report was observed within a session.

Interview and questionnaire data regarding the presence or absence of episodic reports using the past tense are provided (Fig 1). The first episodic report always appeared in the questionnaire data before it appeared in the interview data. Specifically, when questionnaires submitted in one interview included a description of an episodic report in the past tense, children never used the past tense to report an episode within that interview. However, they never failed to do so in the next interview. The first episodic reports to appear in the questionnaire and interview data are also provided (Table 4). According to the questionnaire and interview data, all children, with the exception of TS at 3;4 (interview data), reported additional episodes after the session in which the first episodic report occurred. Milestone 1 was identified as the period between the first episodic report noted by the mother and the first episodic report observed during an interview. Thus, Milestone 1, as assessed using a combination of interview and questionnaire data, emerged at 1;10–3;4 for all the children except KO, for whom it occurred at 3;10–4;0 (Table 5).

**Table 4:** The first episodic reports to appear in the questionnaire and interview data for each child.

	Earlier evidence noted by mother	First evidence in the interview
HM	He said, "Poo (his friend's nickname) ate an Indian strawberry" when asked about the nursery walk. (2; 4)	HM saw a fan and reported: "I played with it (a different fan) at Grandpa (‘s home during a recent visit)." (2; 6)
TS	When asked about a hospital, he said, "My eyes hurt (during a recent visit to eye doctor)." (2; 6)	When asked about recent nursery events, he said "I threw (beans) at Demon (a character)." (He participated in this event for the first time.) (2; 8.5)
KO	When he saw Playland's name on TV, he said, "I visited it!" (3; 10)	When asked about recent nursery events, he said, "Teacher threw beans (at Demon character)." (He participated in this event for the first time.) (4; 0)
YA	When asked about the visit to Playland, he said, "I shook hands with Blue Superman (a character)." (2; 11.5)	He said, "I went to a post office!" (He indeed went there with his mother in the morning several hours before the interview.) (3; 4)
KS	When asked about his brother's birth, he said, "I said to him (immediately before the birth), come out, come out soon." (2; 10)	When he saw a drawing of a snake, he said, "I saw it on Doraemon (an animated program)." (3; 2)
KN	When she came back home from a shrine park and was asked about what she did there, she said, "I met a pussy! (a cat)." (1; 10)	She said, “I picked flowers with Grandma” when she saw flowers outside the window. (2; 0.5)
SA	As she walked along a street with Mom, she said, "I walked here (this street), before." (2; 10.5)	She said, "Bun’s (friend's name) mama brought rice balls (to her home for the first time)” when she heard the phrase “Bun’s mother” in an unrelated context. (2; 11.5)
AH	She said, "I passed my home, (during) the walk" when asked about the nursery walk. (2; 7)	When she saw a figure of Ultraman (a character), she said, "I saw it on TV." (She had rarely seen the program but has recently seen it once.) (2; 11)
MH	When she came back home from outside and was asked about what she did, she said, "I played with Mai (friend's name)." (2; 10.5)	When asked about movies, she said, "I saw Godzilla (movie)." (3; 1)

**Table 5:** The ages of emergence of Milestones 1, 2, and 3 for each child.

	First episodic report (Milestone 1)	First verbal recognition (Milestone 2)	Memory-verb acquisition (Milestone 3)
HM	2 y, 4 m–2 y, 6 m	3 y, 2 m	3 y, 9 m
TS	2 y, 6 m–2 y, 8.5 m	3 y, 10.5 m	4 y, 4 m
KO	3 y, 10 m–4 y, 0 m	4 y, 0 m	4 y, 0 m
YA	2 y, 11.5 m–3 y, 4 m	3 y, 11.5 m	3 y, 9.5 m
KS	2 y, 10 m–3 y, 2 m	3 y, 9 m	3 y, 11 m
KN	1 y, 10 m–2 y, 0.5 m	3 y, 1 m	3 y, 5 m
SA	2 y, 10.5 m–2 y, 11.5 m	3 y, 2 m	4 y, 1.5 m
AH	2 y, 7 m–2 y, 11 m	3 y, 8.5 m	3 y, 11 m
MH	2 y, 10.5 m–3 y, 1 m	3 y, 7 m	3 y, 11 m

Milestone 1, when a child acquires forms of verbal expression of the past (the past tense), was the first of the three developmental milestones for all children. The probability that Milestone 1 would appear alone as the first of the three milestones in all nine children was statistically significant (binomial test, *p* < .01. When the possibility that two or three milestones might occur during the same period was considered, the probability that Milestone 1 would appear alone as the first milestone by chance was 3/13).

### Milestone 2: age at first verbal recognition

**Fig 2:**
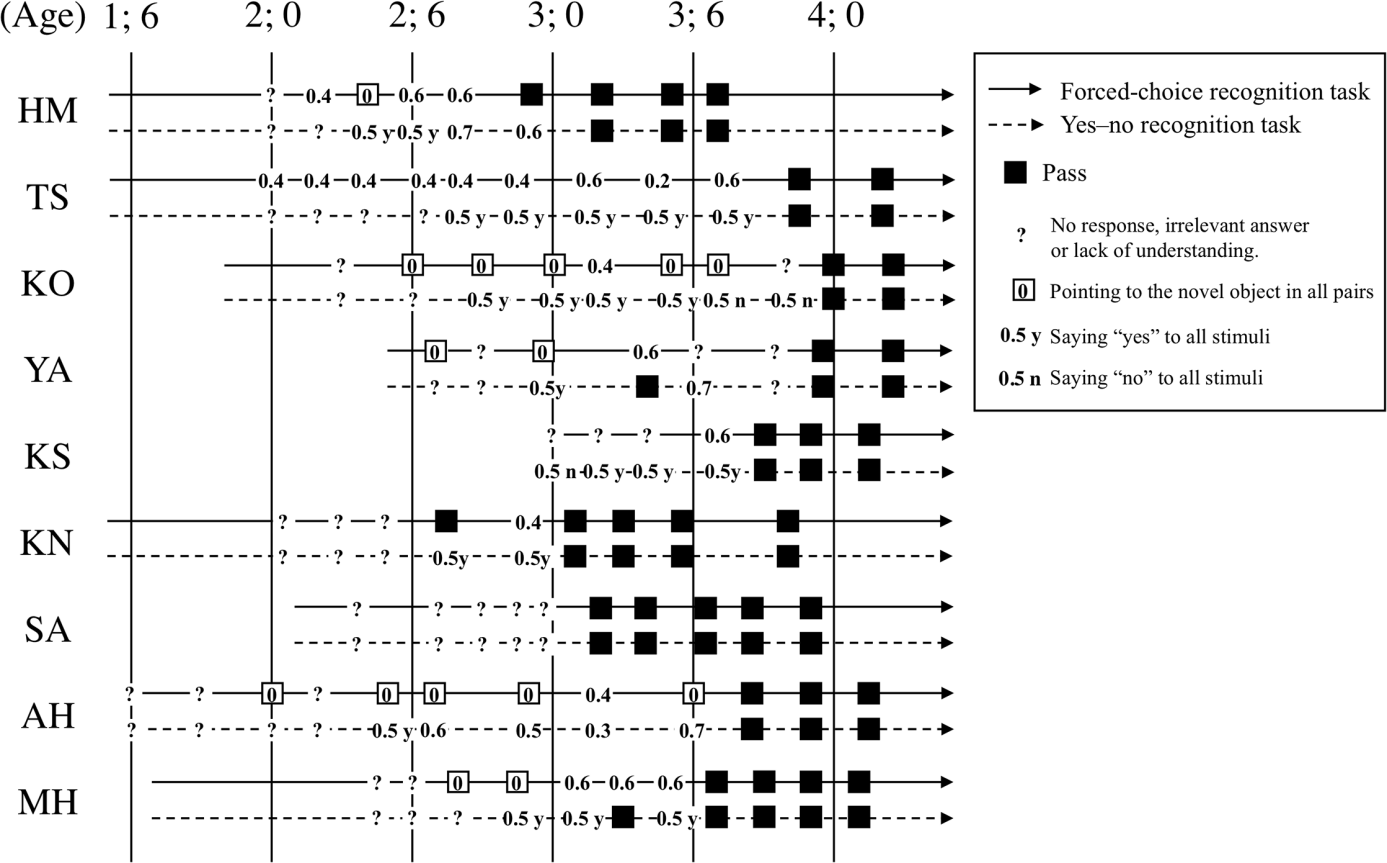
Responses to the forced-choice and yes–no recognition tests as a function of time. The left tail of the arrow represents the age of initial participation. Numbers indicate correct response rates. “■” indicates when the memory tests were passed. In the forced-choice recognition test, a pass was operationally defined as choosing the target drawing in all five drawing pairs while in the yes–no recognition task a pass was defined as correctly stating “Yes” or “No” in response to nine of the 10 single drawings. Idiosyncratic response patterns, such as pointing at the novel object for all pairs in the forced-choice test that asked respondents to point at the old object or saying “Yes” or “No” to all stimuli in the yes–no test, are also noted by the symbols “□,” “0.5y,” and “0.5n,” respectively.

The first verbal recognition milestone (Milestone 2) was identified as the age at which children consistently passed both recognition memory tests (forced-choice and yes–no recognition tests). All nine children reached Milestone 2 between 3;1 and 4;0 (Table 5); performance on the two memory tests is provided (Fig 2). Five children achieved success on the forced-choice and yes–no recognition tests on the same day, two children succeeded on the forced-choice test before succeeding on the yes–no test (HM, KN), and the others (YA, MH) succeeded in the reverse order. No child failed either memory test after succeeding on both memory tests.

Failures in the recognition tasks were characterized by three distinct features. First, nearly all children frequently offered incorrect and/or irrelevant answers. Irrespective of the question posed, children responded by pointing at all drawings, silently looking at drawings, or pointing to (and naming) a drawing without context until 3;0. After 3;0, some children said “I don’t know” (see participants KO, YA, and KS; Fig 2). Second, during a certain period of time, more than half of the children always pointed to the novel object when asked to point to the old object in the forced-choice test (see participants HM, KO, YA, AH, and MH; Fig 2). Third, for a certain period of time, all children except SA claimed that they either had seen all the objects already or had seen none of them in the yes–no recognition test. With the exception of the aforementioned instances, the only perseveration errors, such as always selecting the left (or right) objects in the forced-choice recognition test, were made by TS before 3;0. These inappropriate behaviors disappeared after Milestone 2 was reached.

These idiosyncrasies and their abrupt disappearance suggest a qualitative distinction between unsuccessful and successful responses. Indeed, no participant had a correct response rate of 0.8, and a correct response rate of 0.7 was observed in only three sessions. With these few exceptions, children either had a correct response rate of 0.6 or less in the two memory tests before reaching a certain age or subsequently exhibited a correct response rate of 0.9 or 1.0 in a consistent manner. In conjunction with the many irrelevant idiosyncratic behaviors noted above, these data suggest that children could either successfully recognize all target stimuli or could not recognize any of the stimuli. This dramatic difference indicates that being identified as reaching Milestone 2 did not depend on the difficulty of the task set by the experimenter and was identified without any confounding effects of decay attributable to memory storage.

### Milestone 3: age at memory-verb acquisition

**Fig 3:**
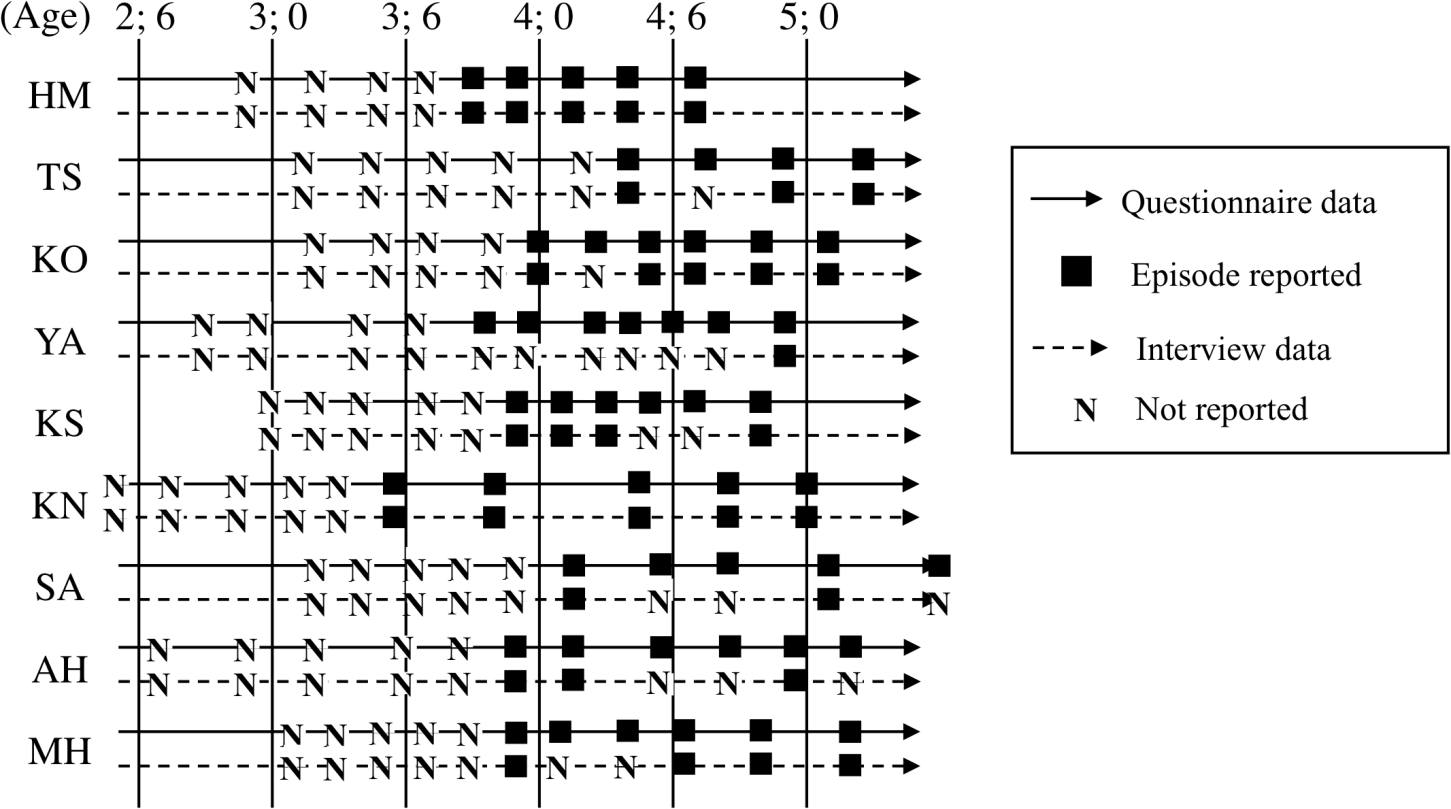
Observation of spontaneous use of memory verbs (“remember” and/or “forget”) in the questionnaire and interview. “■” indicates that evidence of use was observed and “N” indicates that such evidence was not observed.

Data depicting the presence or absence of a memory verb (“remember” or “forget”) in the questionnaire and interview sessions are provided (Fig 3). The first spontaneous use of the memory verbs by each child, with the exception of YA, appeared at the same time point in the questionnaire and interview data. The first time each child used memory verbs during an interview (for YA in the questionnaire data) is provided (Table 6). For seven children (TS, KO, YA, KS, SA, AH, and MH in Fig 3), it was unclear whether the children continued to spontaneously use these verbs during interviews conducted after their first clearly spontaneous use. Psychological verbs such as “remember,” “forget,” and “think” are less frequently used by Japanese-speaking adults than by English-speaking adults during speech [38, 39]. Thus, the relative paucity of spontaneous mentions of these verbs during time-limited interviews is not surprising. However, mothers always noted the use after the first occasion. The first spontaneous use observed in both the questionnaire and interview data was identified as the time of memory-verb acquisition for each child (with the exception of YA, whose questionnaire data alone were used for this purpose).

**Table 6:** The first use of a memory verb during an interview by each child.

	First occurrence
HM	When asked what type of cake he had eaten in a recent event, he responded, “I have forgotten." (3; 9)
TS	When asked about goldfish at his house, "I remember. I scooped up the goldfish." (4; 4)
KO	When Mom could not recall the play event at kindergarten, he said, "You have forgotten!" (4; 0)
YA	When asked about camp, he responded, “I have forgotten." (3; 9.5)
KS	When asked about horse riding, he said, "I do not remember." (3; 11)
KN	KN responded, “I do not remember” when asked who was with her when she got a particular mask. (3; 5)
SA	She said, “I do not remember” when asked about what gifts she received for her third birthday. (4; 1)
AH	When talking about making pancakes with Mom, she said, "Oh, you remember, too!" (3; 11)
MH	When asked about the visit to the lake, she responded, "I remember, hot springs (there)." (3; 11)

Memory-verb acquisition (Milestone 3), which was assessed using a combination of interview and questionnaire data, was achieved at 3;5–4;4 and was the last of the three milestones to be achieved for seven children (Table 5). For these seven children, Milestone 1 appeared first, Milestone 2 appeared second, and Milestone 3 appeared last. The probability that this developmental order (Milestone 1, Milestone 2, and Milestone 3) would be observed in seven of the nine children was statistically significant (binomial test, *p* < .01. The probability that this developmental order would be achieved by chance was 1/13; this was calculated by considering the possibility that two or three milestones might appear during the same period). KO achieved Milestones 2 and 3 at the same age, and YA achieved Milestone 3 about 2 months earlier than he achieved Milestone 2. These cases are not surprising because verbal judgments about recognition and memory-verb use are based on reportable conscious awareness about the past.

To summarize, the first milestone reached was the ability to express autobiographical memories using the past tense (Milestone 1) which was followed by the ability to recognize and verbalize items that had been presented in the past (Milestone 2) and then by acquisition of memory verbs (Milestone 3). The two latter stages tended to appear in this chronological order, although they could also occur simultaneously or in close proximity to each other in either order.

### Individual time course of longitudinal recall in relation to the three milestones

**Fig 4:**
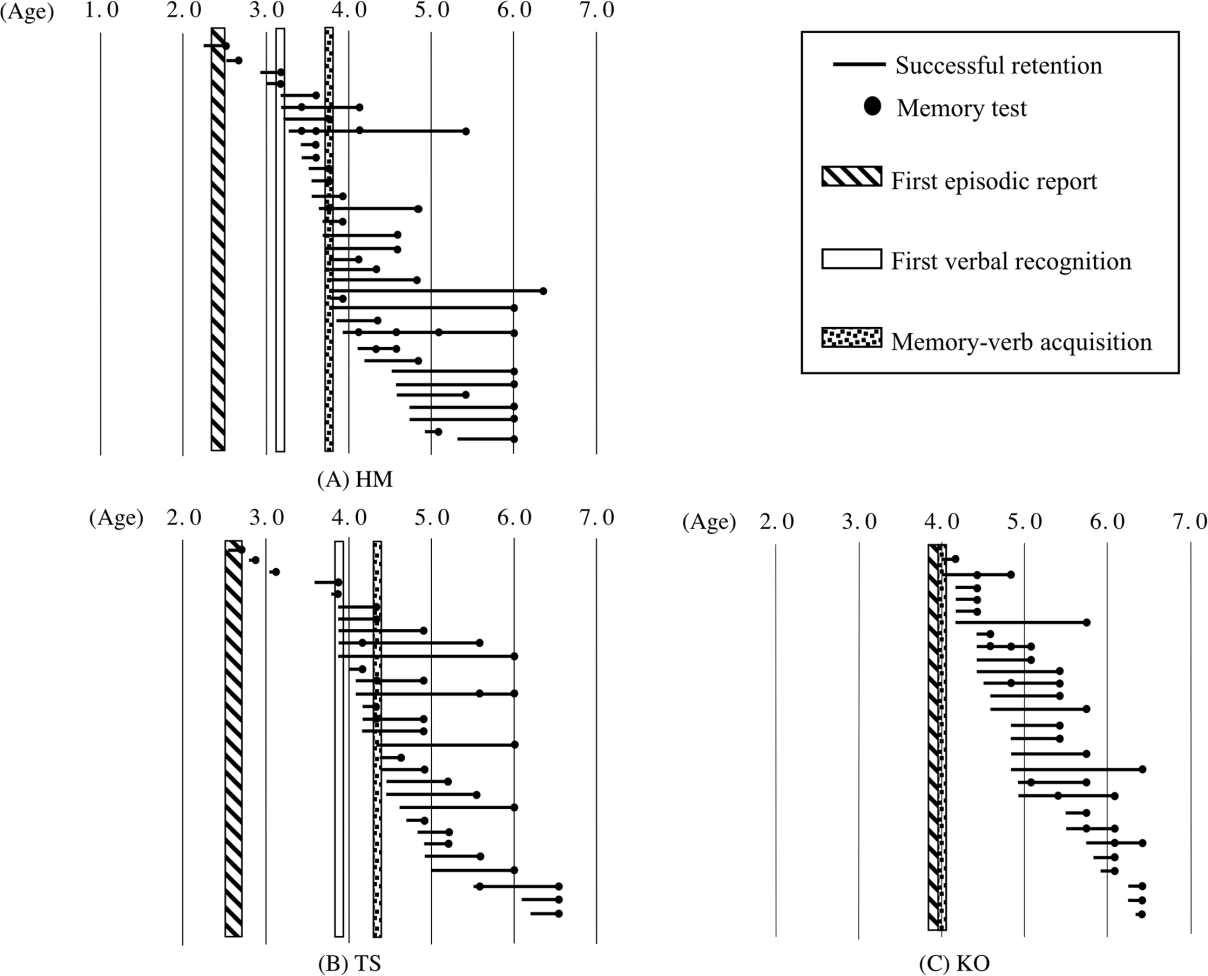
The relationship between the age at experience and successful recall for three children. Each line denotes the successful recall of each event where the left side of the line represents an event during an interview or a daily experience and the black dot refers to the day of the memory test. The age at first episodic report, first recognition, and memory-verb acquisition are superimposed as vertical stripes. (A) Participant HM, (B) Participant TS, and (C) Participant KO.

**Fig 5:**
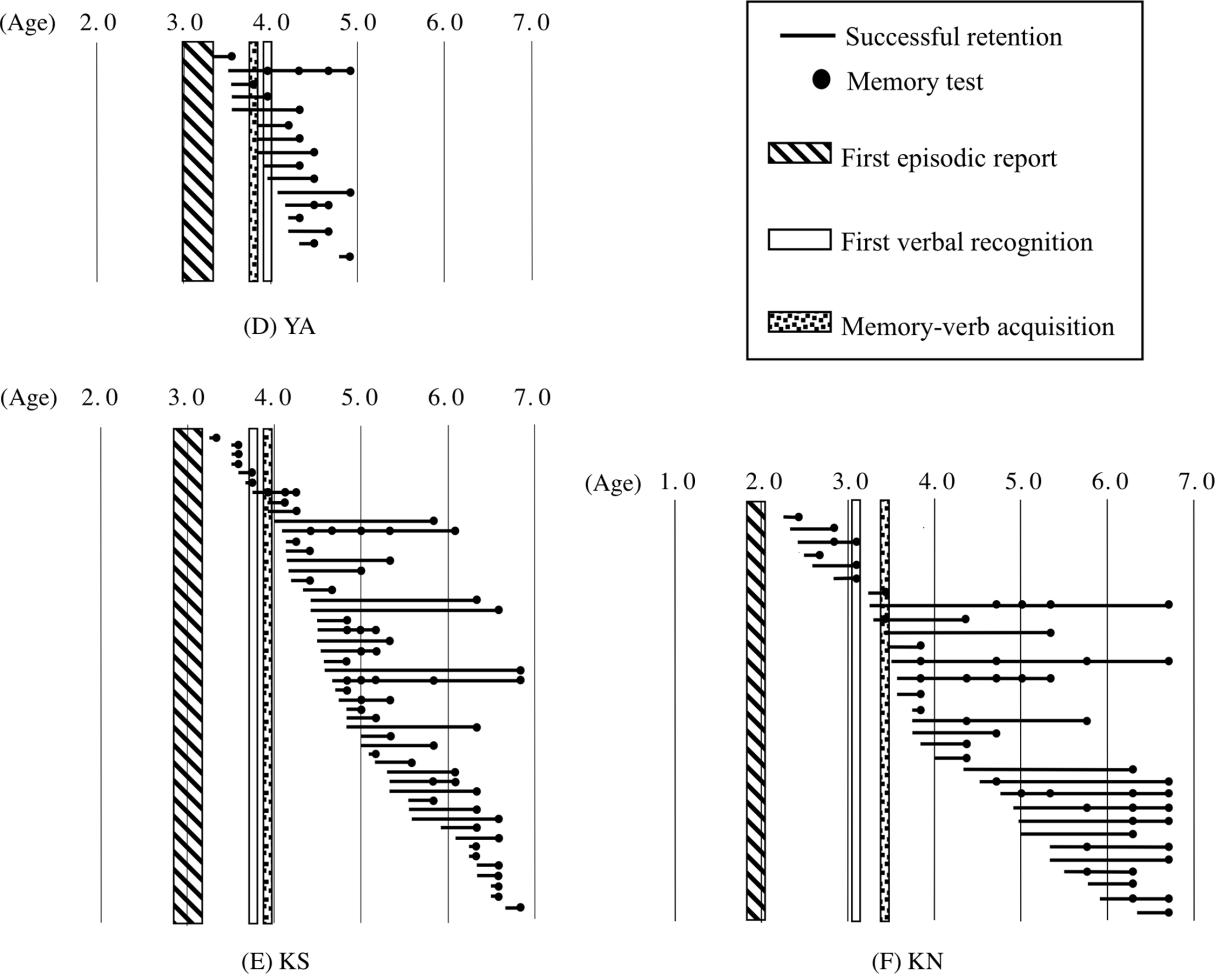
The relationship between the age at experience and successful recall for three children. The symbols are the same as those used in Fig 4. (D) Participant YA, (E) Participant KS, and (F) Participant KN.

**Fig 6:**
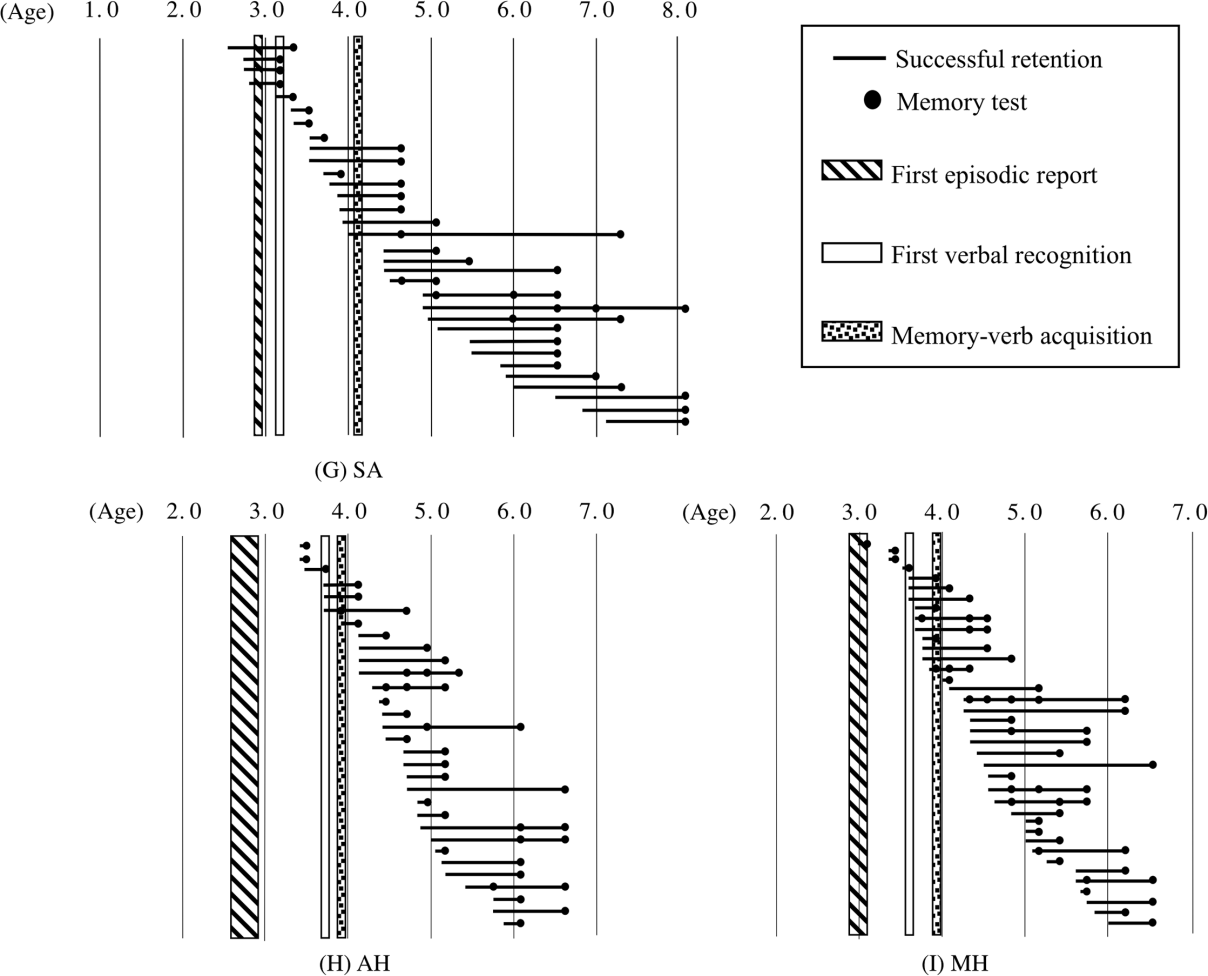
The relationship between the age at experience and successful recall for three children. The symbols are the same as those used in Fig 4. (G) Participant SA, (H) Participant AH, and (I) Participant MH.

All data regarding the successful recall of events that occurred more than 1 month earlier are presented (Figs 4–6), indicating the ages at which the event and the assessment occurred for each child, with the three milestones superimposed on the figures. The successfully recalled event is depicted as a line, with the left side representing the day of the experience and the black dot referring to the day of memory test; thus, the length of the line denotes the retention time. None of the lines whose left sides begin before 3 to 4 years of age extends to 4 years of age or later, meaning that the influence of infantile amnesia can be seen in these data. Until reaching Milestone 2, all children except YA and KO were able to report on a few episodes that they had experienced more than 1 month prior to the assessment. However, after reaching Milestone 2, they no longer had memories of these early events. KO could not recall any events experienced more than 1 month earlier until 4;0, which was the age for both Milestones 2 and 3. KO also never recalled these early events after he became 4;0.

To elucidate the detailed relationships among the dates of the events that were subsequently successfully recalled and the ages at the three milestones, statistical analyses were performed based on the rate of the successful recall of events that occurred during the relevant period; the rate was obtained by dividing the number of events experienced during the period, such as the period before Milestone 2, and recalled successfully during testing (represented as lines in figures) by the number of events addressed in the test during the relevant period (e.g., the period after Milestone 2). The numbers of events addressed during each session are shown in Tables 2 and 3 according to the milestone with which they were associated. The lines with more than one dot were counted according to the numbers of dots (i.e., they were counted both as successfully recalled events and as questions asked). A significantly lower proportion of events experienced before Milestone 2 could be recalled after Milestone 2 compared with the proportion of events that occurred after Milestone 2 and could be recalled after Milestone 2 for all children except YA (HM: *χ* ^2^(1) = 35.3, *p* < .01, φ = 0.63; TS: *χ* ^2^(1) = 37.1, *p* < .01, φ = 0.76; KO: *χ* ^2^(1) = 32.1, *p* < .01, φ = 0.68; KS: *χ* ^2^(1) = 54.8, *p* < .01, φ = 0.67; KN: *χ* ^2^(1) = 40.1, *p* < .01, φ = 0.69; SA: *χ* ^2^(1) = 41.7, *p* < .01, φ = 0.63; AH: *χ* ^2^(1) = 37.1, *p* < .01 φ = 0.68; and MH: *χ* ^2^(1) = 46.2, *p* < .01, φ = 0.73). These results indicate that the age around Milestone 2 is the onset of infantile amnesia.

The possibility that Milestone 3 represents the onset of infantile amnesia was also examined. The ages of the children at Milestones 2 and 3 were the same, or closely approximate, for four children (KO, KS, YA, and AH). For YA, the boundary age of 3;6.5 was closer to Milestone 3 than to Milestone 2. YA barely recalled the events experienced prior to 3;6.5 after that age (the proportion of events experienced before 3;6.5 that were recalled after 3;6.5 was significantly lower than that experienced on or after 3;6.5 and recalled after 3;6.5, *χ* ^2^(1) = 27.7, *p* < .01, φ = 0.72). However, the proportion of events experienced on or after Milestone 2 but before Milestone 3 and then recalled after Milestone 3 was not significantly lower than the proportion of events experienced on or after Milestone 3 and recalled after Milestone 3 for the other children (HM: *χ* ^2^(1) = 3.68, *p* > .05; TS: Yates's correction, *χ* ^2^(1) = 1.79, *p* > .05; KN: Yates's correction, *χ* ^2^(1) = 1.08, *p* > .05; SA: Yates's correction, *χ* ^2^(1) = 1.39, *p* > .05; MH: Yates's correction, *χ* ^2^(1) = 0.01, *p* > .05). This indicates that Milestone 3 was less responsible for the onset of infantile amnesia than was Milestone 2.

Few indicators that Milestone 1 was responsible for the onset of infantile amnesia were found. Although the ages at Milestones 1 and 2 were the same or close for two children (KO and SA), the proportion of events experienced before Milestone 1 (the interview at which the first episodic report was observed) that could be recalled after Milestone 1 but before Milestone 2 was not significantly lower than the proportion of events experienced on or after Milestone 1 and recalled after Milestone 1 but before Milestone 2 among the other children (HM: Yates's correction, *χ* ^2^(1) = 0.14, *p* > .05; TS: Yates's correction, *χ* ^2^(1) = 1.33, *p* > .05; YA: Yates's correction,*χ* ^2^(1) = 1.29, *p* > .05; KS: Yates's correction, *χ* ^2^(1) = 3.54, *p* > .05: KN: Yates's correction, *χ* ^2^(1) = 0.44, *p* > .05; AH: Yates's correction, *χ* ^2^(1) = 1.13 *p* > .05; MH: Yates's correction, *χ* ^2^(1) = 0.55, *p* > .05).

Furthermore, the proportion of events experienced prior to the age of Milestone 2 and retained for more than 1 month before the age of Milestone 2 was significantly lower than the proportion of events experienced and retained for more than 1 month after Milestone 2 among all children except YA (HM: *χ* ^2^(1) = 18.6 *p* < .01, φ = 0.47; TS: *χ* ^2^(1) = 41.9, *p* < .01, φ = 0.68; KO: *χ* ^2^(1) = 37.8, *p* < .01, φ = 0.71; KS: *χ* ^2^(1) = 22.0, *p* < .01, φ = 0.45; KN: *χ* ^2^(1) = 22.9, *p* < .01, φ = 0.53; SA: *χ* ^2^(1) = 12.1, *p* < .01, φ = 0.41; AH: *χ* ^2^(1) = 32.1, *p* < .01 φ = 0.61; MH: *χ* ^2^(1) = 45.6, *p* < .01, φ = 0.68). These findings indicate not only that the age around Milestone 2 is the onset of infantile amnesia but also that it is more difficult to retain events for more than 1 month before the age at Milestone 2 than it is after the age at Milestone 2.

In summary, the data indicate that some critical period for later recall (i.e., events experienced earlier than the critical period that were never recalled after this boundary) was located around Milestone 2.

## Discussion

The present study is the first to provide firm empirical evidence that the achievement of three linguistic milestones in the development of autobiographical memory follows a specific order. These milestones are the age at the *first episodic report* (Milestone 1, at around 2–3 years) when a child first uses the past tense to report on an event that occurred in the past, the age at the *first verbal recognition* (Milestone 2, at around 3–4 years) when a child verbally distinguishes between old and new items, and the age at *memory-verb acquisition* (Milestone 3 at around 4 years) when a child begins to use the verbs “remember” and/or “forget.” Previous studies have reported that children seem to begin to speak about past events after approximately 2 years of age [40, 41] and have examined through tasks whether young children clearly understand the meanings of memory verbs [42, 43], but the ages of the first episodic report, first verbal recognition, and memory-verb acquisition have never been empirically demonstrated.

It may seem improbable that the first episodic report (Milestone 1) precedes the first verbal recognition (Milestone 2) as a recall test is more difficult than a recognition test for adults. However, episodic reporting in the sense used here should be distinguished from the usual definition of episodic recall used for adults [16, 25, 37, 44]. Episodic reports during the period before Milestone 2 may lack meta-cognitive consciousness about remembering a specific past event. In other words, the same inability of children that was shown when they were asked to understand and respond to verbal recognition questions may make their episodic reporting different from adults’ episodic reports. It is not unlikely that the development of episode-reporting skills precedes that of cognitive judgments concerning recognition.

Some might ask why general language assessments were not made in this study. I did not use them because measures of general language ability are reportedly invalid for describing cognitive skills relating to the onset of infantile amnesia [18, 45, 46]. One of the main questions about infantile amnesia is why we can rarely recall events experienced around 2–3 years of age or before, despite the fact that children of this age have sufficient language ability to express the events and are able to report fragmentary details of episodes experienced several months before [9, 24, 37, 47]. If language abilities were fundamentally related to infantile amnesia, events experienced around 2–3 years of age should later be recalled more easily, and the age of the earliest memories for many adults and children should be around 2 years of age. However, such a relationship is not found. On the other hand, it is clear from the past literature regarding child memory that some meta-cognitive linguistic abilities concerning memory would be required for children to report an autobiographical memory and to have consciousness about recalling, and it is thus reasonable to assume that such abilities should be more related to infantile amnesia than to general language skills. Therefore, to invest the limited session time in more promising measurements of specific linguistic skills, I focused on three milestones as potentially more sensitive linguistic indices for cognitive abilities related to infantile amnesia rather than on general language skills. With no standard Japanese-language measures for these memory-related linguistic skills, I decided to test each of them through the reliable method previously established by Uehara [32, 33]: (1) to test whether the child used the past tense and memory verbs by examining mothers' responses to questionnaires and recorded data of all utterances during the interviews and (2) to test whether the child understood the recognition questions in two memory tasks conducted in every interview.

All three milestones have specific implications. Milestone 1 is not meant to be a measure of when a child comes to have consciousness of remembering the past but rather a measure of when the child acquires some sense of the past and the verbal means for expressing it. Young children gradually develop the ability to report personal episodes but they do not often spontaneously report past events without adult-provided cues [16, 37, 44]. Even children as old as 6 years of age often need cues to recall events [16]. This means that a child’s ability in episodic reporting can hardly be judged objectively from the appearance of children’s reports about past events. Instead, the use of the past tense when reporting a past event (Milestone 1) provides a more objective measure of the acquisition of a basic reporting skill about past events, an important component of memory-related linguistic skill.

The first verbal recognition (Milestone 2) is important because one of the conditions necessary for the reportable consciousness of one’s own memories is understanding that one is being asked about a past event. This milestone marks the point at which a child develops the ability to consciously judge whether he or she has previously encountered an event and to provide a correct response when asked about this event. Because the recognition task was designed to test judgment about memory, good performance would be impossible without the child’s awareness of recalling the past in which events were experienced.

When a child acquires memory verbs (reaching Milestone 3) and becomes able to use them appropriately, the child is considered to have reached the developmental stage of comprehending one’s own internal mnemonic processes, meaning he or she is able to have consciousness about memory as well [48, 49]. Some previous research has treated the spontaneous use of these verbs in Japanese as an index of understanding their meaning [50, 51]. Thus, this milestone is a useful index reflecting a child’s ability to monitor his/her own memory states, another important component of memory-related linguistic skill.

Further longitudinal assessment of children’s recall of distant events demonstrated that after Milestone 2, children typically began to forget events that they had experienced prior to their being able to respond properly to recognition questions about episodic memory in the recognition tasks, despite their having had correctly recalled those events prior to reaching Milestone 2. If this beginning of forgetting is called the onset of infantile amnesia, Milestone 1 can be said to occur well before the appearance of this phenomenon. Milestone 2, on the other hand, almost unequivocally coincided with the onset of infantile amnesia. Thus, skills that involve more than simple linguistic abilities and that directly reflect the development of meta-cognitive awareness of personal memory may play an important role in infantile amnesia. A child’s ability to have consciousness about recalling the event may enable him or her to consolidate and recall the event for a longer period of time. Prior to the development of this ability, children may not be able to structure events as complete episodes in their memory, and thus, such events may be unlikely to be recalled later as consciousness about newer memory develops.

It is important to consider the relationship between the present study and past studies. First, Peterson and colleagues [19–21] and Cleveland and Reese [23] indicated, through multiple instances of testing children up to 5 years old, that 5-year-olds had limited recall about events experienced before 2 years of age, whereas they could normally recall events experienced after the age of 2 years, suggesting that 5-year-olds can recall memories from ages younger than suggested in the present study. This age difference may be due to differences in the methods used. These previous studies examined children’s memory of a limited number of events that had been repeatedly discussed, and thus this method may have enabled the children and/or their mothers to predict that the child would be asked about the event in the next test session, which may have facilitated the child’s retention. In contrast, the present study examined memory of about 50–100 events and asked the children about several of them, chosen randomly in each test so that neither the children nor their mothers were able to predict which event would be discussed in each test session. Second, Simcock and Hayne [18, 45] indicated that a preverbal event could not be translated into language after linguistic skills were better developed 6 or 12 months later and that children (between 24 and 48 months of age) with better language skills retained more memories one day after the event than did those with poorer language skills. In contrast, Morris and Baker-Ward [46] observed that 2-year-olds’ preverbal memories could be translated into words 2 months later. Bauer and colleagues [8] repeatedly presented 1–2-year-olds with a model that consisted of multiple procedural sequences, as often used in delayed-imitation tasks, and tested the children’s memory retention multiple times. Their results indicated some effect of verbal elaboration on maintaining memories in children aged 3–4 years or younger. However, their purpose and methods, especially the method of deliberate multiple exposure used by Bauer and colleagues, were radically different from the approach in this study and no direct relationship between their findings and infantile amnesia were indicated in those studies. That said, their results regarding the relationship between verbal abilities and memory in young children aged 3–4 years or younger are consistent with the memory talk of this study’s participants during the period before the onset of infantile amnesia (Milestone 2). Third, some recent studies indicated that the lower boundary age of one’s earliest memory might shift upward with one’s age. For example, Peterson et al. [7] found that the age of the earliest memory of 6–9-year-olds was several months earlier than that of older children. Peterson et al. [52] found that when children's three earliest memories were assessed twice (2 years apart), the lower boundary age shifted by several months between the two tests. Tustin and Hayne [53], who examined the age distribution of the earliest memory, suggested that 5–20-year-olds might be able to recall earlier events than the past literature indicated. However, considering the vast literature on infantile amnesia together with these recent results, the difference in the lower boundary age is not so great among participants of various ages [2, 5, 6]. In contrast to past literature, which has never provided empirical evidence for a single developmental milestone triggering the onset of infantile amnesia, this study demonstrates that one developmental milestone coincides with the onset of infantile amnesia in young children. Taken together, a plausible developmental course for memory reporting skills in relation to infantile amnesia is as follows: The child maintains an ability to fragmentarily recall events experienced several months before until the age of Milestone 2; after acquiring the ability to consciously recall past events, the child has difficulty recalling events experienced before the age of Milestone 2, marking the initial accelerated forgetting. Eventually, this is followed by the second curve of accelerated forgetting, which affects events experienced between 3–4 and 7 years old [11, 12], with the child exhibiting decreasing memory of this period as s/he becomes older.

Several studies conducted using a cross-cultural perspective have indicated that cultures and spoken languages influence the age of infantile amnesia and the development of the ability to make autobiographical reports [54, 55]. However, only a small difference was found in the ages at which Japanese- and English-speaking adults and elementary school children developed infantile amnesia [6, 56]. Thus, although cultural or linguistic differences in the content and structure of autobiographical reports may exist [55, 57], the present results may not be restricted to Japanese-only speakers.

The present findings suggest that the mechanisms underlying infantile amnesia do not merely rest on simple language skills but instead involve more conscious meta-cognitive skills. However, whether infantile amnesia in young children continues into adulthood and how the present results are related to the development of other candidate abilities suggested in previous literature [14–18] remain unknown. Longitudinal investigations of personal memories and cognitive developmental changes throughout the lifespan may expand current knowledge in this regard.

## Supporting Information

S1 Appendix Part of the questionnaire on memory and language development. (PDF)
