# Supplementary material for: Developmental Changes in Memory-Related Linguistic Skills and Their Relationship to Episodic Recall in Children
Source: PLoS One. 2015 Sep 2;10(9):e0137220. doi: 10.1371/journal.pone.0137220 (PMC4558057; doi:10.1371/journal.pone.0137220)
Supplement: S1 Appendix — (PDF) [file pone.0137220.s001.pdf]

Appendix. Part of the questionnaire on memory and language development.

# Have you ever heard your child say the word “remember"?	Yes or No
If so, please provide examples with details.	
# Have you ever heard your child say the word “forget"?	Yes or No
If so, please provide examples with details.	
# Have you ever heard your child talk about his/her own experiences using the past tense?	Yes or No
If so, please describe the style in which s/he talked about them and provide examples with details.<br>1. When your child saw a cue or heard cue words given by you, s/he talked about an episode related to the cue.<br>For example, when the child saw a grape, s/he said, “The grape in the frozen yogurt tasted good." I then asked to the child “Which yogurt?" and s/he said, “Grandma's."<br>2. Your child talked about one of his/her experiences in the absence of any cue, just as adults do in daily conversation.<br>For example, when my child came home, s/he told me, “(Friend’s name) fell down at the nursery.”	
# Please record any significant daily experiences below.	
